# The SMART Safety: An empirical dataset for evidence synthesis of adverse events

**DOI:** 10.1016/j.dib.2023.109639

**Published:** 2023-10-04

**Authors:** Shiqi Fan, Tianqi Yu, Xi Yang, Rui Zhang, Luis Furuya-Kanamori, Chang Xu

**Affiliations:** a MOE Key Laboratory of Population Health Across Life Cycle (Anhui Medical University), Anhui, China; b Anhui Provincial Key Laboratory of Population Health and Aristogenics, Anhui Medical University, Anhui, China; c Department of Maternal, Child and Adolescent Health, School of Public Health, Anhui Medical University, Anhui, China; d Université Paris Cité, Research Center of Epidemiology and Statistics (CRESS-U1153), INSERM, Paris, France; e UQ Centre for Clinical Research, Faculty of Medicine, University of Queensland, Brisbane, Australia

**Keywords:** Evidence synthesis, Adverse events, Randomized clinical trials

## Abstract

Evidence synthesis serves an important role to promote informed decision-making in healthcare practice. A key issue of evidence synthesis is the approach to deal with rare adverse events and the methods to address bias of harm effects. Empirical data is essential to help methodologists and statisticians to solve the issues in evidence synthesis of adverse events. For this reason, we have established SMART Safety dataset, the largest empirical dataset of meta-analyses of adverse events. The dataset contains 151 systematic reviews with 629 meta-analyses on safety outcomes, which covers more than 2,300 randomized controlled trials and 362 harm outcomes, with 10,069 rows and 45 columns of trial level information. All information was double- or even quadra-checked and further verified by referring the original source (e.g., the full-text of the included randomized trials) to ensure high validity of the data.

Specifications Table

**Table:** 

Subject area	Clinical epidemiology, evidence synthesis
More specific subject area	Evidence-based medicine
Data format	Raw Analyzed
Type of data	.xlsx format for the table data
Data collection	Systematic reviews of randomized controlled trials published between January 2015 and January 2020 that focused on adverse events were searched via PubMed [1]. Characteristics (e.g., design, registration) and aggregated data (i.e., event count and group size) of each trial for each specific harm outcomes were collected. As a result, we identified 151 systematic reviews with 629 meta-analyses on safety outcomes, which covers more than 2,300 randomized controlled trials and 362 harm outcomes that consist of the SMART safety dataset with 10,069 rows and 45 variables. All data were double-checked and verified from the original source for potential errors.
Data source location	Institution: Anhui Medical University City: Hefei, Anhui Country: China Latitude and longitude for collected data: 31.84836 N and 117.26305 N, Altitude: 37.51 msl
Data accessibility	Data are available at https://osf.io/g3mdu. DOI: 10.17605/OSF.IO/G3MDU
Related research article	Xu C, Yu T, Furuya-Kanamori L, et al. Validity of data extraction in evidence synthesis practice of adverse events: reproducibility study. BMJ. 2022; 377: e069155.

## 1 Value of Data

• The database comprehensively compiled all available information of included randomized controlled trials from 151 systematic reviews on safety outcomes.
• The database adopted a scrupulous data collection strategy to minimize human errors, with all collected data being triple or even quadruple checked after double extraction. This guarantees the reliability of the data.
• These data will be beneficial for healthcare provider interested in investigatig the harm effect of a specific drug, which covers more than 500 types of durgs and 362 subjective and objective adverse events.
• The dataset can be utilized for exploring the evidence synthesis methodologies, as its data structure encompasses both study-level and meta-level information.

## 2 Data Description

Evidence synthesis serves an important role to promote informed decision-making in healthcare practice [1]. A key issue of evidence synthesis is the approach to deal with rare adverse events and the methods to address bias of harm effects [2]. Empirical data is essential to help methodologists and statisticians to solve the issues in evidence synthesis of adverse events. For this reason, we established the SMART Safety dataset.

SMART Safety is an empirical dataset for drug safety of randomized controlled trials. The dataset was established by collecting information of randomized controlled trials from systematic reviews of medication-related harms that identified via PubMed [3]. The final dataset consists of 151 systematic reviews with 629 meta-analyses for harms, which covering over 2,300 randomized controlled trials. There levels of information were available in the dataset:

• Information of systematic reviews, which include: the name of the review author, topic of the review, region of the review author, number of trials included, and registration details.
• Information of the meta-analyses, which include: the safety outcomes of each meta-analysis, type of the outcomes classified by 1) objective or subjective, 2) composite or non-composite.
• Information of the randomized trials, which include: name of the first author, year of publication, journal of publish, event counts of related adverse events and number of participants per intervention group, intervention details (e.g., drug type, control, dosage, mean treatment duration), source of funding, registration details, population age (child, adult, elder), region of the trials.

**Fig. 1:**
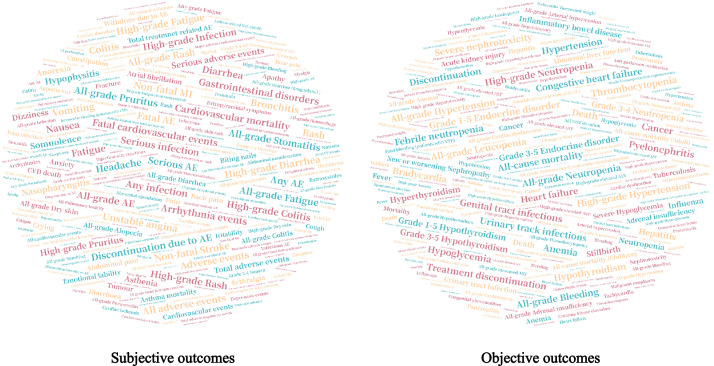
Word cloud analysis of safety outcomes (subjective or objective).

**Fig. 2:**
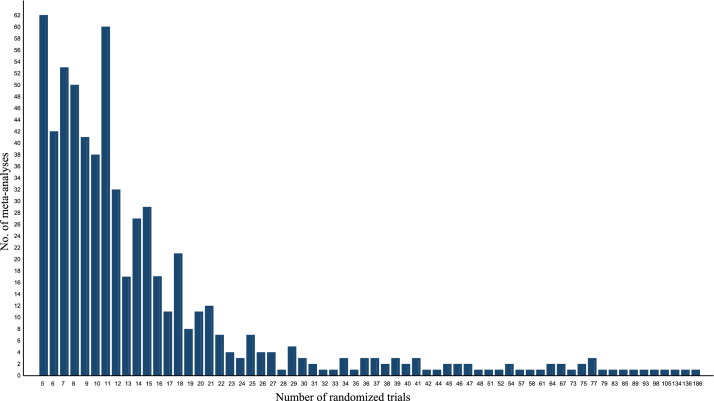
Number of randomized controlled trials per meta-analysis.

The basic information of systematic reviews was presented in Table 1. In brief, the majority of the reviews were conducted by authors whose primary affiliation is in Asia (45.03 %), Europe (26.49%), and America (21.19%). The number of studies included in each review ranged from 5 to 195, with a median value of 16 (IQR: 10 to 26). With regard to the topics, cancer (45.03%), osteoarticular diseases (10.60%), diabetes (7.28%), and cardiovascular diseases (5.30%) were the four most frequently researched diseases. There were 362 harm-related outcomes in total, and Fig. 1 illustrates the Word cloud analysis of harm-related outcome categories in 629 meta-analyses. Our dataset includes 629 meta-analyses with a range of 5 to 186 randomized controlled trials each, as shown in Fig. 2. The data profile and supplementary code book provides more details of the information.

**Table 1:** Basic characteristics of eligible systematic reviews and trials.

Basic characteristics	Summary
**Region of corresponding author** (review level)	*N* = 151
Africa	9 (5.96%)
Americas (North and South)	32 (21.19%)
Asia	68 (45.03%)
Europe	40 (26.49%)
Oceania	2 (1.32%)
**Number of trials included** (review level)	16 (IQR: 10 to 26)
1 to 9 (minimum was 5)	36 (23.84%)
10 to 29	83 (54.30%)
30 or more (maximum was 195)	33 (21.85%)
**Topic of disease** (review level)	*N* = 151
Cancer	68 (45.03%)
Diabetes and cardiovascular diseases	19 (12.59%)
Osteoarticular diseases	16 (10.60%)
Others	48 (31.79%)
**Registration** (study level)	*N* = 10,069
Yes	7,483 (74.32%)
No	2,408 (23.91%)
Missing	178 (1.77%)
**Center** (study level)	*N* = 10,069
Multiple centers	7,891 (78.37%)
Single center	350 (3.48%)
Missing	1,828 (18.15%)
**Funding** (study level)	*N* = 0,069
Industry	8,440 (83.82%)
Industry & academic	108 (1.07%)
Academic	737 (7.32%)
No funding	3 (0.03%)
Missing	781 (7.76)
**Publication type of study** (study level)	*N* = 10,069
Article	9,848 (97.81%)
Abstract	18 (0.18%)
Registration only (unpublished)	198 (1.97%)
Non-RCT	5 (0.05%)
**Accessible of full-text** (study level)	*N* = 9,848
Yes	9,598 (97.46%)
No	250 (2.54%)

RCT: randomized controlled trial.

## 3 Experimental Design, Materials and Methods

### 3.1 Search strategy

On July 28, 2020, an information specialist developed the search strategy and conducted the literature search on PubMed for systematic reviews and meta-analyses of medication-related harms from January 1, 2015, to January 1, 2020. The representativeness of the literature search was verified by using related systematic reviews from 2008 to 2011 that identified in multiple databases (e.g., PubMed, Embase, CENTRAL[3]) and searching these systematic reviews on PubMed, where an estimated coverage of PubMed of these systematic reviews ranging from 93.9% to 99.3%. The search strategy was presented in the supplementary Table S1.

### 3.2 Inclusion criteria

Systematic reviews and meta-analyses of randomized controlled trials that treated medication-related harms as the exclusive outcomes were of interested to be included. In addition, to be included in the dataset, systematic reviews should have at lead one meta-analysis that compares the risk of harms of one medication intervention to active drugs or inactive controls (e.g., placebo, waitlist), and the meta-analysis should have at least five randomized controlled trials, with the 2 by 2 table data were available for each trial. We limited meta-analyses should have five and more trials to reduce potential random error since one of our sub-projects was to investigate the statistical performance of current evidence synthesis methods [4]. We set requirement on the 2 by 2 table data since it allows us to identify any potential data extraction errors and take steps to solving the errors [5].

We defined harms as ‘*any adverse medical events occurring in patients or subjects during clinical practice*’, which could be an adverse effect, adverse reaction, harm, or complication associated with any healthcare intervention [6]. We defined systematic review and meta-analysis on the basis of the article title as stipulated by the review authors, where a systematic review often involves more than one meta-analysis, with each meta-analysis refers to one specific outcome (e.g., vomit).

### 3.3 Literature screening and data collection

Two authors reviewed the records independently by the titles and abstracts (stage 1) and then the full texts (stage 2), utilizing the Rayyan (https://www.rayyan.ai/) web-application. This application allows us to screen the records in a blinded pattern. Only the records excluded by both authors simultaneously in stage 1 were considered excluded; and the remaining records were further screened in stage 2, with all discrepancies resolved by consensus. Finally, a total of 151 systematic reviews were identified, of which, 629 meta-analyses amongst these reviews met the inclusion criteria.

Data collection was implemented by two groups of team researchers, again, in a double-extraction pattern (i.e., two groups, collect data independently). Two types of information were involved during the data collection:

1) Information that could be directly extracted without additional judgment. This include: name of the review author, the publication year of the review, the outcomes of the review, the name of each trial, the 2 by 2 table data for each trial, analytic rules (e.g., intervention-to-treat), the name of intervention and control, median treatment duration of each arm, dosage of the intervention, population age of each trial (children, adult, elderly), source of funding of the trial, number of centers of the trial, registration status of the trial, and region of the trial.
2) Information that required some degree of assessment and judgement. This include: the risk of bias of each trial and type of outcomes of each meta-analysis.

For the first type of data, human errors may involve during the data collection process, all of the information were further checked after the double extraction process by referencing the primary data sources (i.e., full texts, appendices, trial registrations), and necessary correction was made when errors were identified (see details in Supplementary file). With regard to trial registration, the information of registration may not be reported in the trial publications; Attempts were made by searching the following four registries based on the name of the principal researcher, name of the intervention and control: ClinicalTrials.gov, World Health Organization's International Clinical Trials Registry Platform, European Clinical Trials Register, and International Standard Randomized Controlled Trial Number (ISRCTN) registry [7,8]. We matched the trials based on sample size, intervention measures, and publications listed in the registry. Only trials for which registration information was absent from both sources were considered non-registered trials.

Human errors and even measurement bias would involve in the second type of data as well. Therefore, to eliminate potential error or bias, the assessment or judgement were all based on two groups of team researchers independently. The type of outcome was judged by two senior methodologists of clinical epidemiology, and their decisions were compared through a blind review process by a third person. Risk of bias was assessed by two groups of team researchers (group 1: FY, YY, TQ; group 2: XY, RZ) bias using an adapted RoB 2.0 [9] with emphasis on applicable components and domains, which include: 1) random sequence generation, 2) allocation concealment, 3) blinding of participants, 4) blinding of healthcare provider, and 5) blinding of outcome assessor. Disagreements were then discussed until consensus was reached.

## 4 Limitations

There were some limitations that merit consideration in the SMART safety database. Firstly, the database is hampered by the absence of pertinent information within some RCT reports or the unavailability of full-text documents, resulting in a range of missing data from 1.05% to 27.54%, thereby affecting the overall comprehensiveness of the dataset. In addition, the data we have collected is in the form of summarized data, rather than individual participant data (IPD). Consequently, for studies requiring IPD, this database cannot provide the requisite information. Furthermore, our search was restricted in terms of safety outcomes and publication years, this limitation could potentially impact the representativeness of the included studies.. Finally, the database exclusively focuses on pair-wise comparisons, which involve the assessment of two interventions. Therefore, this database might not be applicable for methodological studies requiring multiple intervention comparisons, such as Network Meta-Analysis.

## Ethics Statement

We confirm that participant data has been fully anonymized and comply with the PubMed platform's data redistribution policies.

## CRediT authorship contribution statement

**Shiqi Fan:** Writing – original draft. **Tianqi Yu:** Investigation, Validation. **Xi Yang:** Investigation, Validation. **Rui Zhang:** Investigation, Validation. **Luis Furuya-Kanamori:** Conceptualization, Methodology. **Chang Xu:** Conceptualization, Investigation, Validation, Writing – review & editing.

## Data Availability

SMART Safety (Original data) (OSF).

## Appendix Supplementary materials

Image, application 1

Image, application 2
