# Supplementary material for: The SMART Safety: An empirical dataset for evidence synthesis of adverse events
Source: Data Brief. 2023 Oct 4;51:109639. doi: 10.1016/j.dib.2023.109639 (PMC10589771; doi:10.1016/j.dib.2023.109639)
Supplement: Supplementary file 1 [file mmc1.pdf]

Supplementary Table S1. Elaborate search strategy within PubMed

Search steps	search strategy
1	"Systematic Reviews as Topic"[Mesh] OR "Systematic Review" [Publication Type] OR "Meta-Analysis as Topic"[Mesh] OR "Meta-Analysis" [Publication Type] OR "meta-analysis"[Title/Abstract] OR "meta analysis"[Title/Abstract] OR "systematic review"[Title/Abstract]
2	"randomized controlled trials as topic"[MeSH Major Topic] OR "clinical trials as topic"[MeSH Major Topic] OR "controlled clinical trials as topic"[MeSH Major Topic]
3	"randomized controlled trial*"[Title/Abstract] OR "controlled clinical trial*"[Title/Abstract] OR "clinical trial*"[Title/Abstract] OR "controlled trial*"[Title/Abstract] OR "trial*"[Title/Abstract]
4	"safety"[Title/Abstract] OR "harm*"[Title/Abstract] OR safe[Title/Abstract] OR poisoning[Title/Abstract] OR toxicity[Title/Abstract] OR tolerability[Title/Abstract] OR "complication*"[Title/Abstract] OR "adverse event*"[Title/Abstract] OR "adverse outcome*"[Title/Abstract] OR "untoward effect*"[Title/Abstract] OR "side effect*"[Title/Abstract] OR adverse n2 reaction[Title/Abstract]
5	#2 or #3
6	#1 AND #4 AND #5
7	Protocol[Title] OR overview [Title] OR "narrative review" [Title]
8	#6 NOT #7
9	(#8) AND (("2018/01/02"[Date - Publication]: "2020/01/01"[Date - Publication])) AND (humans[Filter]) Filters: Humans
10	(#8) AND (("2015/01/01"[Date - Publication]: "2018/01/01"[Date - Publication])) AND (humans[Filter]) Filters: Humans
11	#9 or # 10

Supplementary Table S2. Contribution of the dataset and quality control

Information	Contributors	Comparison (Blind)	Double checking	Triple checking	Quadruple checking
**Original information extracted from systematic reviews, randomized trials, and registration platforms**					
Aggregated data (events, group size)	Chang Xu, Tianqi Yu, Xiaoqin Zhou, Hanming Dai	NA (Self-Checking)	Tianqi Yu	Chang Xu	Xi Yang
Treatment duration (Median)	Chang Xu, Tianqi Yu, Xiaoqin Zhou, Hanming Dai	NA	Chang Xu, Tianqi Yu	NA	NA
Funding	Chang Xu, Tianqi Yu, Xiaoqin Zhou, Hanming Dai	NA	Chang Xu	NA	NA
Registration	Yueyuan You	NA	Fengying Zhang	NA	NA
Results posting	Yueyuan You	NA	Fengying Zhang	NA	NA
Interventions (Two arms)	Chang Xu, Tianqi Yu, Xiaoqin Zhou, Hanming Dai	NA	Xi Yang	Rui Zhang	NA
Dose	Chang Xu, Tianqi Yu, Xiaoqin Zhou, Hanming Dai	NA	Yuan Tian, Xing Xing	Yuan Tian, Xing Xing, Xi Yang	Chang Xu
ITT/PP	Xi Yang	NA	NA	NA	NA
Age	Xi Yang	NA	NA	NA	NA
Risk of bias	**Group 1:** Fengying Zhang, Yueyuan You, Tianqi Yu<br>**Group 2:** Xi Yang<br>(Group 1 and Group 2 were independent for assessment)	Rui Zhang	Xi Yang, Rui Zhang	Xi Yang, Rui Zhang	NA
Center and Region	Fengying Zhang, Yi Zhu	NA	Fengying Zhang	NA	NA
DOI	Chang Xu, Tianqi Yu, Xiaoqin Zhou, Hanming Dai	NA	Fengying Zhang	NA	NA

Generated information based on original information					
SRID, MAID	Tianqi Yu	NA	Chang Xu	Chang Xu	Chang Xu
Outcome type (objective/composite)	Chang Xu, Luis Furuya-Kanamori (Blinded)	Rui Zhang	Chang Xu, Luis Furuya-Kanamori	NA	NA
Error and Error Type	Chang Xu, Tianqi Yu	NA	Chang Xu, Tianqi Yu	NA	NA
Subgroup	Chang Xu, Tianqi Yu	NA	Chang Xu, Tianqi Yu	Xi Yang	NA
Assessable of Full-text	Chang Xu	NA	Rui Zhang	NA	NA
Net Interventions	Xi Yang	NA	Rui Zhang	NA	NA

All contributors: Chang Xu, Tianqi Yu, Xi Yang, Fengying Zhang, Hanming Dai, Rui Zhang, Yueyuan You, Yuan Tian, Xing Xing, Xiaoqin Zhou, Luis Furuya-Kanamori, Cuncun Lu, Ke Ju, Yi Zhu

Supplementary Table S3. Missing data information of SMART Safety dataset.

Variables with missing data	Missing counts	Proportions
**Events in treatment group (r1)**	774	7.69%
**Events in control group (r2)**	792	7.87%
**Group size in intervention group (n1)**	106	1.05%
**Group size in control group (n2)**	121	1.20%
**Treatment duration in intervention group (t1)**	1,367	13.58%
**Treatment duration in control group (t2)**	1,436	14.26%
**Dose of net intervention**	479	4.76%
**Interventions**	310	3.08%
**Controls**	396	3.93%
**Age (child, adult, eld)**	369	3.66%
**Concealment**	471	4.68%
**Blind for participants**	471	4.68%
**Blind for care provider**	471	4.68%
**Blind for outcome assessor**	471	4.68%
**Access of full-text**	471	4.68%
**Center information**	1,828	18.15%
**Region information**	2,773	27.54%
**Funding**	781	7.76%
**Registry**	179	1.78%

Supplementary Table S4. Records of data collection.

11-April, 2021	Draft the protocol
14-April, 2021	Changing “follow-up time” to “treatment time” after consulting experts of clinical trials.<br>**Reason:** Any treatment has wash-out period, after the period, any adverse events may not be caused by intervention.
17-April, 2021	Send for collaborators for reviewing, no context changes
22-April, 2021	Distinguishing the treatment duration and control duration in data extraction form based on extraction training
25-April, 2021	Adding more information in secondary outcome, say, the seven “Whether…”, as an effort to see how well the harmful effects were investigated
4-May, 2021	Change inclusion criteria: meta-analysis of RCTs to meta-analysis of clinical trials.<br>Reason: Some meta-analyses claimed they included phrase II or II trials, and did not mention whether these were RCTs or not. Based on our experience, the majority were RCTs. Therefore, we expand the inclusion criteria from RCTs to clinical trials.
15-May, 2021	During the data extraction, we noticed many of the systematic reviews failed to report or conduct the risk of bias of each trial. After an online meeting, we decided to assess the risk of bias by our research team.
17-May, 2021	Two assistants were added to the research, with a background in evidence-based medicine. They take charge of the assessment of risk of bias. Since the assessment of ROB is somewhat subjective, the two assistants are blinded; they were not and will not be informed of the aim of the project and the potential comparisons. And the assessment of risk of bias will be started after the finish of the data extraction (possibly in July).
28-May, 2021	Change in the analysis of the main outcomes: We primarily plan to limit

	RCTs with 1:1 design to facilitate the estimation of the ORs in the case of zero-events. But then after our recent simulation study, we found continuity correction works well even when the ratio ranges from 0.51 to 1.99, therefore we “relaxed” the limits to 0.51 to 1.99.
1-June, 2021	We primarily collected data of the clinical trials from the systematic reviews, while for the sample we finished (which accounted for 1/3 of the total), we found that many of the data were incorrectly recorded by these systematic reviews. Based on an urgent online meeting (CX and TQ), we decided to re-do the data extraction, directly from the original studies.
5-June, 2021	Online meeting with a pharmacist about the definition of different treatments/controls.
10-June, 2021	Some clinical trials may have a flexible treatment schedule, for example, they use A drug with 5 mg at week 1, and 10 mg at week 2, and 20 mg at week 3, and then continue 20 mg for the following 11 weeks.<br>Makes it difficult to extract the dose. After a discussion (CX and TQ), we decided to use the maximum dose in our study.
14-June, 2021	Add the tools that will be used for ROB assessment.
15-June, 2021	Add information for extraction: whether the trials involve two periods, and the design (double/single blind, open label) of each period.
26-June, 2021	Two assistants (WJ, FY) are ready to start the assessment of the ROB. An online meeting is held to discuss the use of ROB 2 (XC, TQ, WJ, FY). Prior training for the two assistants based on 5 trials is prepared and will start tomorrow. The second online meeting will be held after the training for a further discussion of any issues during the preliminary assessment.<br>**Note:** We finished about 50% of the systematic reviews for the intended data collection, and the included trials of these systematic reviews are ready for the ROB assessment. For the rest 50%, we may finish it 3 weeks later, and as long as we finish, we will prepare the trials included in these reviews

	for WJ and FY for the ROB assessment.
30-June, 2021	The two assistants finished the first period training of 2 RCTs; there were many disagreements between them on the judgement of each item. Thus, an online meeting is held discussion the issues of the assessment of each domain, each item, by reading the explanation of the ROB 2. After 1 hour's discussion, an agreement is achieved of the assessment criteria. And the two assistants will continue to assess the remaining 3 RCTs for training.
2-July, 2021	The two assistants finished the remaining 3 RCTs, and an online meeting is held to discuss further issues they met during the assessment. There is a high agreement on the judgement of the items this time, and only 3 minor points are involved. The training is now finished, and they are ready to formally start the assessment of the ROB.
15-July, 2021	During the data extraction of the meta-analysis data from original RCTs, we recorded many pieces of incorrect information from these meta-analyses. We then decided to treat this as an additional project that investigated how many meta-analyses with the data were incorrect. In this additional project, we will add systematic reviews other than drug/biologics treatment in order to increase the representativeness of the dataset. Both the data obtained from the meta-analyses and the data obtained from original RCTs will be recorded. We will first investigate the proportions that systematic reviews failed to extract the correct 2 x 2 table data; we will also record the type of incorrectness; we will further compare the meta-analysis based on correct data to the incorrect one to see how the incorrection impacts the results. Another assistant (Z.XQ) will take charge of the data extraction for systematic reviews other than drug/biologics treatments. The data will then be double-checked by the assistant after she finishes it.
15-July, 2021	XC and DMH finished the data extraction of SRs assigned to them, and started double-checking the data they extracted.

17-July, 2021	Until now, XC checked for 571 rows of 2 by 2 table data he extracted from RCTs (with 28 meta-analyses), and found 21 rows were incorrect by himself. The main reason is that RCTs present the same outcome for several times, thus leading to confusion (e.g. study by List et al. Diabetes Care. 2009 Apr;32(4):650-7. Table 2. Urinary tract infection). The estimated proportion of mis-extraction by himself is then 21/571=3.7%. Suggests a high accuracy of the data extraction. However, the mis-extraction also suggests the importance of double-checking process. Therefore, an online meeting (XC, YTQ, and DMH) is then held by XC, and everybody is required to check each cell of the 2 by 2 table very carefully to ensure 0% error. In addition, YTQ and DMH are required to report their own accuracy to XC during their double-checking process.
18-July, 2021	Until now, XC checked for 696 rows of 2 by 2 table data he extracted from RCTs (with 43 meta-analyses), and found 24 rows were incorrect by himself. The estimated proportion of mis-extraction by himself is then 24/696=3.4%. The accuracy is better for the data extracted more recently (3.7% vs. 2.4% [3/125]).
20-July, 2021	Until now, XC checked for 850 rows of 2 by 2 table data he extracted from RCTs (with 54 meta-analyses), and found 24 rows (no errors from 697-850) were incorrect by himself. The accuracy is 24/850=2.8%. Again, the accuracy is much better for the data extracted more recently (3.7% vs. 2.4% vs. 0%).
20-July, 2021	Z.XQ finished the meta-analytic data (2 by 2 table for each included study) extraction from the forest plot/tables of the meta-analyses other than drug/biologics. She will start the 2 by 2 table data extraction from original RCTs thereafter, and she was required to double-check the data she extracted from meta-analysis and report her accuracy to XC.
20-July, 2021	Report by DHM: From rows 2 to 519 and row 1068 to 1310, there were 5 to

	10 errors (she estimated) in the data extracted by DHM. The proportion ranges from 5/760 (0.66%) to 10/760 (1.32%).
21-July, 2021	Report by XC: From rows 850 to 1073, there were 12 errors in the data extracted by XC, with a proportion of 12/223=5.38%. This is because he put the data of “any infection” into “serious infection” while the data of “serious infection” into “any infection” in Silvia 2016’s review.
22-July, 2021	Report by XC: From rows 1073 to 1678, there were 4 typo errors in the data extracted by XC, with a proportion of 4/605=0.66%.
22-July, 2021	XC starts to extract the data of the remaining 25 systematic reviews assigned to him.
27-July, 2021	DHM starts to extract the data of the remaining 15 systematic reviews assigned to her.
30-July, 2021	Report by XQ: From rows 1 to 145, there was 1 typo error in the data extracted by her, with a proportion of 1/145=0.69%.
30-July, 2021	TQ starts double-checking for the data she extracted.
5-Aug, 2021	XC has finished the data extraction of the remaining 25 systematic reviews with 86 meta-analyses assigned to him. He plans to check the data thereafter.
7-Aug, 2021	DHM has finished the data extraction of the remaining 14 systematic reviews assigned to her. And she plans to check the data thereafter.
7-Aug, 2021	Report by TQ: Until now, by checking the data she extracted, the proportions of errors were 4 from rows 1 to 373 (4/373=1.07%), 6 from rows 374 to 799 (6/425=1.41%), 4 from rows 800 to 1151 (4/351=1.14%), 5 from rows 1151 to 2351 (5/1200=0.42%), and 1 from rows 2352 to 3435 (1/1083=0.093%).
7-Aug, 2021	TQ starts to extract the data of the remaining 16 systematic reviews assigned to her.
23-Aug, 2021	DHM finished all the SRs assigned to her.

23-Aug, 2021	Report by DHM: For the remaining 14 SRs with 68 meta-analyses, there were about 1-5 errors by herself based on her double-checking. The proportion of errors ranged from 1/1023=0.08% to 5/1203=0.49%.
26-Aug, 2021	Report by XC: For 14 SRs of 39 meta-analyses he double-checked until today, there were 4 errors from 647 rows, with a proportion of 4/647=0.62%. There remain 11 SRs to be double-checked.
27-Aug, 2021	TQ finished the data extraction of the 16 SRs assigned to her, and she will start the double-checking process thereafter.
30-Aug, 2021	Report by XC: For the remaining 11 SRs with 47 meta-analyses, there were 4 errors among 609 rows, with a proportion of 4/609=0.66%. Two for typo errors, and two (one study) for mistakes in the definition of outcome.
2-Sep, 2021	Report by TQ: For the remaining 16 SRs with 59 meta-analyses, there were 6 errors by herself among 1325 rows, with a proportion of self-error of 6/1325=0.45%, mainly due to typo errors as well as failing to find the outcomes in the first time.
19-Sep, 2021	ZXQ finished the data extraction for the 40 SRs based on the original RCTs.
19-Sep, 2021	Report by ZXQ: From the 40 SRs with 2495 rows, there were 22 errors in total by herself, with a proportion of self-error of 22/2495 = 0.88%.
26-Sep, 2021	Data cleaning and checking were finished by the principal author, assisted by the three assistants.
7-Oct, 2021	A fourth-round checking was finished by the principal author, focusing on the information of subgroup analysis, those identified with data extraction errors, type of errors classification, and those without full-text recorded by the four assistants. A few minor typos/confusions were addressed.
16-Oct, 2021	A fifth-round checking by TQ finished, which mainly focused on the coding of the ID of systematic review, ID of each meta-analysis, information of subgroups, and eligibility of all systematic reviews. TQ found that 9 systematic reviews should not be excluded and need further data extraction.

17-Oct, 2021	An online meeting by XC and TQ for the 9 additional systematic reviews, and decided to extract the data by one of the assistants that do not know the summarized results.
18-Oct, 2021	Hanmin started data extraction for the 9 additional systematic reviews.
22-Oct, 2021	Hanmin finished the data extraction of the addition systematic reviews. And XC started double-checking for the data.
25-Oct, 2021	XC finished the double-checking of the data. As such, all the data extractions were finished.
26-Aug, 2021 **(ROB and checking)**	One of the assistants who took charge of the ROB assessment (MW) decided to withdraw from the research group, after 2 months of his participating. The assessment of ROB remains one assistant. The project leader started to recruiting new assistant to continue the assessment of ROB.
13-Jan, 2022	TQ and YY took over the ROB assessment. Considering the large workload, the project leader decided to recruit more assistant, and arrange TQ, FY, and YY as group 1 for ROB assessment, with each take charge of about one-third of the trials.
11-Feb, 2022	FY finished the ROB assessment of her parts. In the point of quality control, the lead author requires FY to re-check the data for ROB assessment.
10-Mar, 2022	TQ finished the ROB assessment of her parts. In the point of quality control, the lead author requires TQ to re-check the data for ROB assessment.
11-May, 2022	FY finished the self-checking of the ROB of her parts.
20-June, 2022	YY finished the ROB assessment of her parts. And now, group 1 finished all the ROB assessments. In the point of quality control, the lead author requires YY to re-check the data for ROB assessment.
21-Aug, 2022	YY finished the self-checking of the ROB of her parts.
27-Nov, 2022	TQ finished the self-checking of the ROB of her parts.
17-Jan, 2023	YX and RPW participant in the research as ROB assessors for Group 2. A pilot training by 5 randomized trials was assigned to them.

25-Jan, 2023	YX and RPW finished the training, and started the ROB assessment (blinded to Group 1).
11-Feb, 2023	RPW withdrawal from the research group. And YX takes charge of all the ROB assessment of Group 2.
12-Mar, 2023	Two undergraduate students (XX and TY) join in the research team; after a pilot training, they were asked to mark the dose information, namely, marked studies with the same dose within each meta-analysis using consecutive numbers. For example, if two studies had the same dose in a meta-analysis, they will be marked with the same number. This process is designed to facilitate the following matching process, and will be done separately by the two students.
23-Mar, 2023	The two students finished the marking task for dose information.
24-Mar, 2023	Under the supervise by YX, the two students cross-check their marked results and discuss any disagreements.
24-Mar, 2023	The discussion meeting by YX, XX, TY found some studies with dose information were ambiguously extracted which make it difficult to determine the dose. After discussed with project leader XC, we decided to check the dose information, and then re-mark the number.<br>As we already have the dose information, the checking process will be done by one student (TY), and finally will be checked again by project leader XC.
13-April, 2023	YX finished the ROB assessment.
13-April, 2023	ZR compared the ROB information assessed by Group 1 and Group 2, and marked the RCTs with inconsistent information.<br>YX and ZR discuss the disagreements and re-check the information from related trails.
16-April, 2023	ZR and YX finished the double-checking of the ROB, and delivered the data to the project leader XC. XC and ZR started to check any missing RCTs, and detected 357 had missing ROB information (in both groups). After

	carefully checking of the dataset, XC confirmed that the missing information was due to the process of removing duplicates and a failing to record the citation form of some RCTs.
16-April, 2023	ZR and YX started to assess the ROB of the remaining 357 RCTs, separately.
17-Apri, 2023	Of the 357 RCTs, ZR and YX obtained full-texts of 318 RCTs.<br>For the remaining 39, CX re-checked the assess of full texts. For these, 10 were identified as conference abstracts, with 2 of which were duplicates. For the remaining 29, CX was able to obtain the full-texts of 14 RCTs. For the rest 16, the full-texts were failed to obtain through many attempts (e.g., asking for help).<br>Therefore, 332 RCTs were to be assessed of the ROB, separately, by ZR and YX. Based on previous experience, about 10 to 20 mins for each RCT of the ROB, thus they planned 50 RCTs per day.
20-April, 2023	Report from ZR: 200 RCTs were finished the ROB assessment.<br>Report from YX: 200 RCTs were finished the ROB assessment.
23-April, 2023	ZR and YX finished the ROB assessment.<br>XC started to check the agreements and disagreements of their information.<br>The marked disagreements were discussed by ZR and YX, based on re-checking of the related RCTs.
24-April, 2023	A study (Gen Dent, 2013, 61: 70-6) without full-text was obtained through internet inquiry and was further identified as non-RCT.
24-April, 2023	ZR and YX finished the double-checking process and reached a consensus on all RCTs of the ROB information.
24-April, 2023	Report from TY: of the 10,069 lines, by now, he has finished 7,237 of the dose information checking and correction. And for the dose information, from line 1 to line 5386 (from the first to 363 meta-analyses) dose information was well extracted. While after then, the dose information was

	not well extracted. TY expected he would finish all the checking and correction by the next two days.<br><br>Note: This is because the total information was extracted by four authors separately, and the information of the first 363 meta-analyses was extracted by the lead author (XC) and DHM. The rest were extracted by TQ, where the dose information was ambiguous.
25-April, 2023	Considering the dose information was well extracted for the first 363 meta-analyses, XC checked the marked information of the first 363 meta-analyses by XX and TY for the most update data.<br>For the 5,386 lines, XC detected 131 lines with errors; the error rate was 2.43% (131/5386). All the detected errors were corrected.
26-April, 2023	Report from TY and XH: the rest 2832 lines of the dose information were checked and updated.<br>XC starts to re-check the dose information and the marked of the dose information (dose indicator).
27-April, 2023	Report from XC: From lines 5387 to 6305, which refers to the 364 to 390 meta-analyses, there were 120 errors in dose indicator, with an error rate of 13.1% (120/919). This is because the original dose information of this part and thereafter (extracted by TQ) lack of courses information that led to a wrong judgement on dose indicator (The information has been further checked and added by TY).
28-April, 2023	Report from XC: From lines 6306 to 7755, which refers to the 391 to 481 meta-analyses, there were 148 errors in the dose indicator, with an error rate of 10.21% (148/1450), with the same reason below recorded.
29-April, 2023	Report from XC: From line 7756 to line 10069, which refers to the 482 to 629 meta-analyses, there were 287 errors in the dose indicator, with an error rate of 12.40% (287/2314). Tile new, XC finished the triple checking of the

	dose indicator information.<br>In total, from line 5387 to line 10069, which refers to the 364 to 629 meta-analyses, there were 555 errors in the dose indicator, with an error rate of 11.85% (555/4683).
