# Supplementary material for: The SMART Safety: An empirical dataset for evidence synthesis of adverse events
Source: Data Brief. 2023 Oct 4;51:109639. doi: 10.1016/j.dib.2023.109639 (PMC10589771; doi:10.1016/j.dib.2023.109639)
Supplement: Supplementary file 2 [file mmc2.pdf]

ILLUSTRATION OF THE VARIABLES

In the SMART safety dataset 45 variables were provided. The meaning of these variables is illustrated below.

Review level:

- **SRID:** The identification number of a systematic review, ordered from 1 to 151. Each systematic review contains 1 or more meta-analyses, which indicated by MAID.
- **SR_author:** The first author of each systematic review .
- **Region of corresponding author:** The region of the corresponding author of the systematic review.
- **Number of trials:** Number of randomized controlled trials included in the systematic review.

Meta-analysis level:

- **MAID:** The identification number of a meta-analysis, ordered from 1 to 629.
- **Outcomes:** The interested outcomes of randomized controlled trials related to drug adverse events included in a meta-analysis.
- **Outcomeindicator:** The interested outcome types: objective or subjective.
- **Compositeendpoints:** The interested outcome types: composite or non-composite.

Study level:

- **R1:** The event counts of specific outcomes in the intervention arm.
- **N1:** The total sample counts of specific outcomes in the intervention arm.
- **R2:** The event counts of specific outcomes in the control arm.
- **N2:** The total sample counts of specific outcomes in the control arm.
- **T1:** The median treatment duration of the intervention arm, unified as week.
- **T2:** The median treatment duration of the control arm, unified as week.

- **Funding:** The funding information obtained in a randomized controlled trial study can be classified into five categories: Industry/Industry-employmer, Industry + Non-profit, Non-profit, No funding, and Not report, which are coded as 1, 2, 3, 4, and 5, respectively. .

- **StudyID:** The identification number of a study (randomized trials) across all meta-analyses. This variable is used to ensure the order of the dataset.

- **Citation:** The citation segment for a study (randomized trials) within all meta-analyses, comprising the journal, year, issue (volume), and page numbers.

- **Year:** The publication year of the manuscript for a study (randomized trials).

- **Registry:** An indicator to distinguish whether the registration of a study (randomized trials): Yes or No.

- **Registration_number:** The registration number of a study (randomized trials).

- **Source:** The source of registration information: Full-text or Searching.

- **Studystartdate:** The start date of a study (randomized trials).

- **Actual primary completion date:** The actual primary completion date of a study (randomized trials)

- **Firstposted:** The first posted of the manuscript for a study (randomized trials).

- **Regis_form:** An indicator to distinguish registration status of a study (randomized trials) : Prospective or Retrospective registration, A trial registered before or within one month of the trial started was regarded as perspective registration, and vice versa. "N" means no registration Information.
- **Regis_time:** An indicator to distinguish whether the registration date of a trial is earlier than the completion date of that experiment: Before, After, Withboth. "N" means no registration Information.

- **Resultsposted:** An indicator to distinguish whether study results were uploaded to the registration platform: Yes or No.

- **Error:** An indicator to distinguish whether data extraction errors in meta-analysis: Yes or No.

- **Type:** The occurrence of data extraction error and the detailed type of errors by the original systematic reviews, which was classified on the base of our recent paper (BMJ. 2022; 377: e069155). There were five types of errors: numerical error, ambiguous error, mismatching error, zero-assumption error, and misidentification error, see detailed definition in our recent paper (BMJ. 2022; 377: e069155). "N" means no errors.

- **Subgroup:** An indicator to distinguish whether existence of subgroup analysis in the same study: Yes or No.

- **SubID:** An indicator to distinguish whether a same study contains multiple intervention groups (mostly with two levels of doses, e.g., penicillin 50 mg, penicillin 25 mg, compared to placebo). Within each meta-analysis, the rows with the same non-zero number of SubID means it belongs to the same study, ordered by Arabic numbers. “0” means there is no multiple intervention. This indicator is used to remove the duplicates, as a study with multiple interventions share the same control (A: penicillin 50 mg vs. placebo, B: penicillin 25 mg vs. placebo), when combining them together, there would be a increased Type I errors (False positive error).

- **Dataaccessible:** An indicator to distinguish whether data from a study (randomized trials) is accessible: Yes or No.

- **Dose_indicator:** The identification number for the same dose in all studies (randomized trials).

- **Net_i:** The net intervention drugs in the intervention group

- **Net_c:** The net control drugs in the control group

- **c_indicator:** An indicator to distinguish the presence or absence of a net control, Yes or No.

- **Ittpp:** The type of analysis chosen for data analysis, including ITT, mITT, PP, mPP.

- **Age:** The age grouping of participants in the randomized trials: Child, Adult, Old.

- **Fulltext:** An indicator to distinguish whether a study (randomized trials) has a fulltext.

- **Concealment:** An indicator to distinguish whether a study (randomized trials) has concealment: Yes, Probably Yes, No and No Information.

- **Blind_patient:** An indicator to distinguish whether a study (randomized trials) has patient blinding: Yes, Probably Yes, No and No Information.

- **Blind_care:** An indicator to distinguish whether a study (randomized trials) has medical interveners blinding: Yes, Probably Yes, No and No Information.

- **Blind_out:** An indicator to distinguish whether a study (randomized trials) has assessor of outcomes blinding: Yes, Probably Yes, No and No Information.

- **Center_ind:** An indicator that distinguishes whether a study (randomized trial) is a single-center or multicenter study.

- **Region:** Region in which the trial was conducted
